# Activation of testosterone‐androgen receptor mediates cerebrovascular protection by photobiomodulation treatment in photothrombosis‐induced stroke rats

**DOI:** 10.1111/cns.14574

**Published:** 2024-02-08

**Authors:** Yu Feng, Zhihai Huang, Xiaohui Ma, Xuemei Zong, Celeste Yin‐Chieh Wu, Reggie Hui‐Chao Lee, Hung Wen Lin, Michael R. Hamblin, Quanguang Zhang

**Affiliations:** ^1^ Department of Neurology Louisiana State University Health Sciences Center Shreveport Louisiana USA; ^2^ Wellman Center for Photomedicine Massachusetts General Hospital Boston Massachusetts USA

**Keywords:** bEnd.3 cells, PBMT, stroke, testosterone, vascular protection

## Abstract

**Rationale:**

Numerous epidemiological studies have reported a link between low testosterone levels and an increased risk of cerebrovascular disease in men. However, there is ongoing controversy surrounding testosterone replacement therapy due to potential side effects. PBMT has been demonstrated to improve cerebrovascular function and promote testosterone synthesis in peripheral tissues. Despite this, the molecular mechanisms that could connect PBMT with testosterone and vascular function in the brain of photothrombosis (PT)‐induced stroke rats remain largely unknown.

**Methods:**

We measured behavioral performance, cerebral blood flow (CBF), vascular permeability, and the expression of vascular‐associated and apoptotic proteins in PT‐induced stroke rats treated with flutamide and seven consecutive days of PBM treatment (350 mW, 808 nM, 2 min/day). To gain further insights into the mechanism of PBM on testosterone synthesis, we used testosterone synthesis inhibitors to study their effects on bEND.3 cells.

**Results:**

We showed that PT stroke caused a decrease in cerebrovascular testosterone concentration, which was significantly increased by 7‐day PBMT (808 nm, 350 mW/cm^2^, 42 J/cm^2^). Furthermore, PBMT significantly increased cerebral blood flow (CBF) and the expression of vascular‐associated proteins, while inhibiting vascular permeability and reducing endothelial cell apoptosis. This ultimately mitigated behavioral deficits in PT stroke rats. Notably, treatment with the androgen receptor antagonist flutamide reversed the beneficial effects of PBMT. Cellular experiments confirmed that PBMT inhibited cell apoptosis and increased vascular‐associated protein expression in brain endothelial cell line (bEnd.3) subjected to oxygen‐glucose deprivation (OGD). However, these effects were inhibited by flutamide. Moreover, mechanistic studies revealed that PBMT‐induced testosterone synthesis in bEnd.3 cells was partly mediated by 17β‐hydroxysteroid dehydrogenase 5 (17β‐HSD5).

**Conclusions:**

Our study provides evidence that PBMT attenuates cerebrovascular injury and behavioral deficits associated with testosterone/AR following ischemic stroke. Our findings suggest that PBMT may be a promising alternative approach for managing cerebrovascular diseases.

## Graphical abstract

Photobiomodulation therapy mitigates cerebrovascular injury, including damage to the blood‐brain barrier, reduced cerebral blood flow, and decreased levels of vascular tight junction proteins, ameliorates the behavioral deficits associated with testosterone following an ischemic stroke.

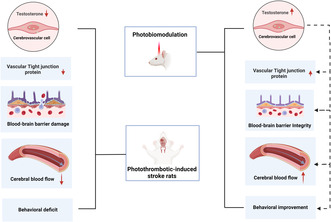

## 1 INTRODUCTION

Ischemic brain stroke is one of the most severe neural disorders and causes lifetime disability worldwide.^1^ Current data indicating an increased incidence of stroke in younger age groups are concerning, because strokes in young individuals have a disproportionately large economic impact since victims are disabled during their most productive years.^2^ Recently, it has been reported that more young women than men have strokes, suggesting possible importance of sex‐mediated etiologies of stroke.^3^ Additionally, low testosterone levels are associated with an increased risk of previous and future ischemic stroke or post‐stroke mortality in men.^4^, ^5^ Animal experiments found that castrated young rats (300–350 g) receiving testosterone before middle cerebral artery occlusion (MCAO) have a significant reduction in infarct volume and an increase in neurogenesis.^6^, ^7^ These results suggest that there is a potential beneficial effect of testosterone after cerebral ischemia in young male rats.

Abnormal cerebrovascular tight junctions (TJ), impaired microvascular perfusion, and reduced cerebral blood flow (CBF) have been detected in ischemic stroke.^8^, ^9^ Testosterone influences the blood‐brain barrier (BBB) properties and function in males.^10^, ^11^ Hypogonadal men given testosterone replacement displayed enhanced cerebral perfusion in the midbrain and superior frontal gyrus.^12^ Castration of adult male mice significantly decreased the expression of TJ proteins, such as Claudin 5 and Zonula occludens‐1 (ZO‐1), in the preoptic area of the hypothalamus, leading to changes in morphological arrangement, along with delocalization and disorganization of brain endothelial TJ proteins.^13^ Experiments *in vitro* also showed that pro‐androgens could augment ZO‐1 and Claudin 3 expressions, and TJ formation in the murine brain microvascular endothelial bEnd.3 cell line.^14^ Testosterone can bind to androgen receptors (ARs) in cerebral blood vessels in smooth muscle cells and the endothelium, to exert biological effects.^15^ Testosterone's beneficial effects on ischemic stroke were blocked by an ARs antagonist.^16^ BBB function could be affected by the androgen levels in the brain via ARs.^17^

Photobiomodulation therapy (PBMT) is an alternative treatment for stroke and traumatic brain injury that inhibits inflammatory responses and neuronal apoptosis while promoting neurogenesis *in vitro* and *in vivo* models of cerebral hypoxia/ischemia.^18^, ^19^, ^20^ PBMT has also been shown to increase regional CBF^21^ in the vertebral arteries and internal carotid arteries, which are the major blood supply to the brain. In addition, PBMT regulates new blood vessel formation during oral wound healing in rats.^22^ However, much less is known about the mechanisms of cerebrovascular protection by PBMT after cerebral ischemia.

Low‐level laser therapy (LLLT) using a 670‐nm diode laser applied to the testes effectively increased in increasing serum testosterone levels.^23^ Steroid dehydrogenase enzymes 17β‐hydroxysteroid dehydrogenase 5 (17β‐HSD5) and 3β‐hydroxysteroid dehydrogenase (3β‐HSD) catalyze the formation of testosterone.^24^, ^25^ One study found PBMT increased steroid dehydrogenase activity in cultured porcine granulosa cells.^26^ However, further validation is needed to confirm and characterize changes in steroid dehydrogenase and testosterone concentrations in the cerebral vessels after PBMT.

Therefore, we examined the impact of testosterone on cerebrovascular function and its involvement in ischemic injury following PBMT in rats with photothrombosis (PT)‐induced stroke, as well as in bEnd.3 cells undergoing oxygen‐glucose deprivation (OGD) treatment. The present study provides a mechanistic link between cerebrovascular testosterone and PBMT. Furthermore, our work identifies cerebrovascular testosterone as a promising molecular target for ischemic stroke treatment.

## 2 MATERIALS AND METHODS

### 2.1 Animals and study design

Animal use and experimental protocols were approved by the Institutional Animal Care and Use Committee of Louisiana State University. all of the experimental procedures were in accordance with guidelines set by the Institutes for the Humane Care and Use of Laboratory Animals in compliance with the ARRIVE guidelines. Two‐month‐old male Sprague‐Dawley rats were used in this study. Based on our experience and preliminary studies, we propose that statistical significance can be achieved with 6‐10 animals/group per group. Upon collection of pilot data, a power analysis (*α* = 0.05, *β* = 0.20, SigmaStat software) was performed to minimize animal use. Animals were housed (2 rats/cage from the same litter) in a temperature‐ and light‐controlled room (23°C with a light/dark cycle of 12/12 h) with food and water available ad libitum. Rats were randomly assigned into the following five groups: (a) Control group (Sham group), (b) Control group which received an intra‐cerebroventricular injection (*icv*) of flutamide through the mini‐osmotic pump (FL group), (c) an experimental group with PT stroke (PT group), (d) an experimental group with PT stroke, which received PBMT (PT + PBM group), and (e) an experimental group with PT stroke, which received PBMT plus an intra‐cerebroventricular injection of flutamide (PT + PBM + FL group). The experimenters were blinded to group assignment.

### 2.2 Preparation of mini‐osmotic flutamide‐releasing pump

Flutamide (94,495, Sigma‐Aldrich, USA, dissolved in 60% DMSO saline) was filled into a mini‐osmotic pump (Alzet Corporation, USA) with a release speed of 208 ng/h for 14 days. Control rats were infused with 60% DMSO (J66650.AK; Thermo Fisher Scientific, USA) saline (the high DMSO concentration needs to be used to fully dissolve flutamide), but this concentration of DMSO did not impair cognitive function.^27^ In this experiment, rats received an intra‐cerebroventricular infusion of flutamide (10 μg)^27^ or vehicle using brain infusion kits (0008663; Alzet Corporation, USA) 7 days before PT‐stroke insult. For ICV injections of Flutamide or the vehicle DMSO, a guide cannula with its stylet, was stereotaxically inserted into the right lateral ventricle of the rats, under anesthesia with carprofen. The stereotactic coordinates were (mm): AP ‐0.8, L ‐1.5, V ‐3.5.^28^

### 2.3 PT stroke injury

The PT stroke model was established following the methodology outlined in our previous study.^19^ In brief, rats were initially anesthetized with isoflurane and securely placed in a stereotactic frame. Subsequently, Rose Bengal dye (0.1 mg/g, administered intraperitoneally) was given to the animals over a 5‐min period prior to exposing the skull. The periosteum was delicately removed, and then the skull was exposed to a 6‐mm diameter cold white light beam from a fiber optic cable for 15 minutes. This light was positioned 1.8 mm anterior to the bregma and 2.5 mm lateral from the midline. Throughout the surgical procedure, the animals were maintained on a heating pad to ensure a rectal temperature of 37 ± 0.5°C. Following the procedure, the skin was closed with wound clips. Analgesia will be administered using carprofen (5 mg/kg, subcutaneous injection) starting at the time of surgery and continuing twice daily for 48–72 h post‐surgery. The animals will be monitored daily following the operation.

Two rat mortalities during the anesthesia process in the PT stroke experiment led to their exclusion from the study.

### 2.4 PBM treatment

**FIGURE 1:**
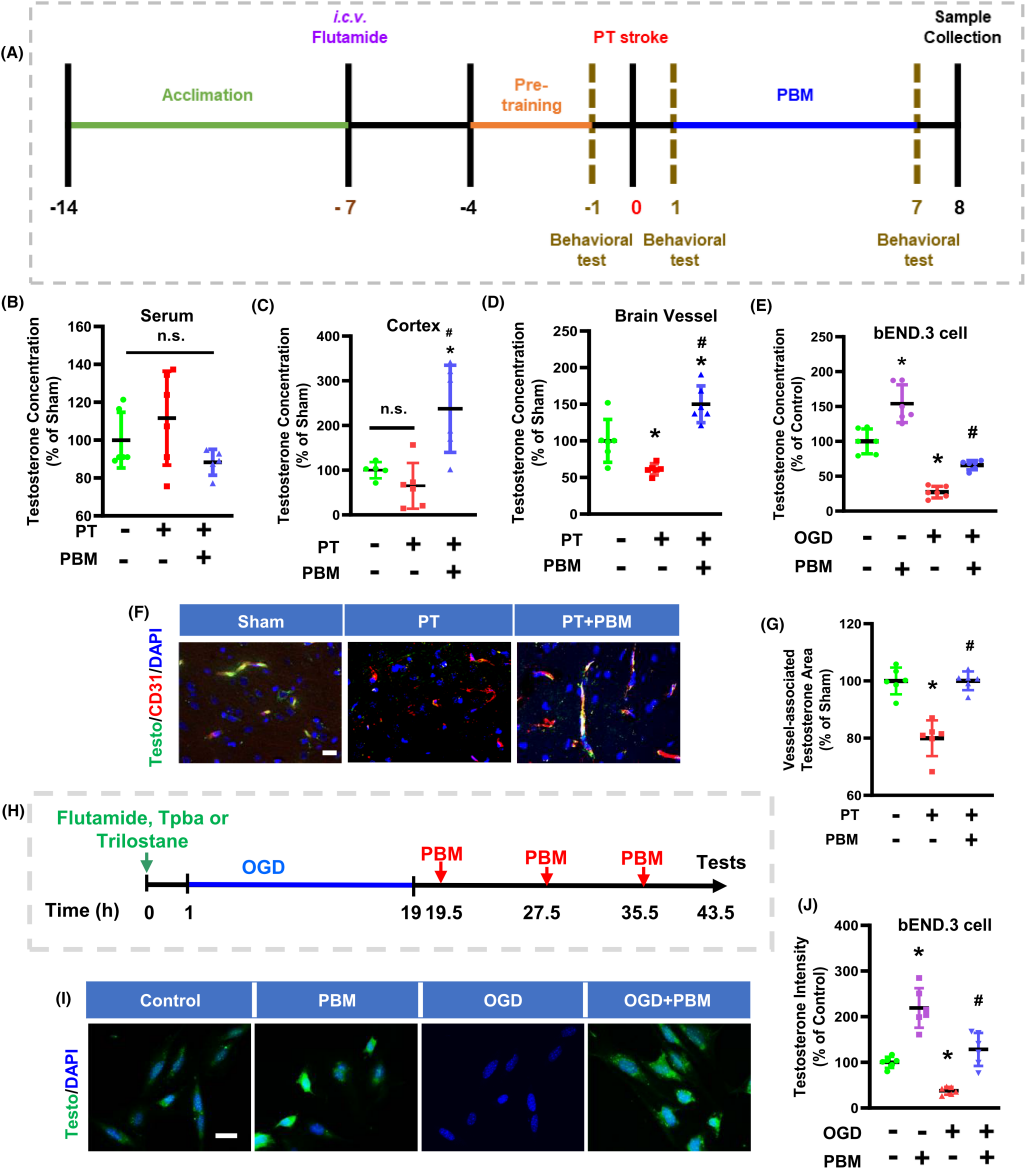
Photobiomodulation treatment (PBMT) increases vascular testosterone concentrations in photothrombosis (PT)‐stroke rats and OGD‐treated bEND.3 cells. (A) Experimental timeline of the rat study. (B–D) Measurements of testosterone concentration in the serum, total protein of peri‐infarct cortex, and samples of cerebral blood vessel. (E) Quantitative analysis of testosterone concentration in bEND.3 cells using an ELISA assay. (F) Representative immunofluorescence staining images of testosterone (green), CD31 (red), and DAPI (blue) in the peri‐infarct area. (G) Quantitative analysis of the colocalization of testosterone and CD31 intensity (ratio to Sham) in the peri‐infarct area. (H) Experimental timeline of studies on bEND.3 cells. (I) Representative immunofluorescence staining images of testosterone (green) and DAPI (blue). (J) Quantitative analysis of testosterone staining in bEND.3 cells. * indicates *p* < 0.05 vs. Sham or Control group; # indicates *p* < 0.05 vs. the PT‐stroke group or the OGD group. All data are expressed as mean ± SD (*n* = 5–6), one‐way ANOVA analysis followed by post hoc Bonferroni's test was used for rats' experiments, two‐way ANOVA analysis followed by post hoc Bonferroni's test was used for cellular experiments. Scale bar for tissue = 20 μm. Scale bar for cells = 10 μm. Testo, Testosterone; OGD, Oxygen‐glucose deprivation.

The detailed procedure for PBMT is illustrated in Figure 1A. As reported in our previous study,^29^ 2‐min daily laser irradiations (808 nm, 350 mW/cm^2^ on the scalp, 25 mW/cm^2^ at cerebral cortex tissue), were applied using a Diode IR Laser System (808 M100; Dragon Lasers) on the area of the scalp underlying the infarct injury from day 1 to day 7. The laser power meters (#FM33‐056; Coherent Inc, USA) were used to measure the power density. During treatment, rats were lightly restrained using a transparent cone (DCL‐120; Braintree Scientific, MA). The distance between the laser tip and the rat scalp was 35 cm, and the laser spot was about 1.5 cm^2^. For the PBM treatment *in vitro*, the bEnd.3 cells were exposed to an irradiance of 25 mW/cm^2^ (Laser spot was 2 cm^2^/well for 24 well plate and 9.6 cm^2^/well for 6 well plate) for 2 min (fluence: 3 J/cm^2^), starting 30 min after OGD and repeated every 8 h for a total of three applications.

### 2.5 Laser speckle contrast imaging

Laser speckle contrast imaging was used to measure CBF. Rats were placed into a stereotaxic frame following anesthesia. A circular cranial window was created by thinning a circular area of the skull using a high‐speed drill (RWD Life Science, China). Regional cortical blood flow was measured by illuminating the infarct area with a 750‐nm laser using the RFLSI III Speckle Contrast Imaging System (RWD Life Science, China). The duration of laser exposure was 5 min. CBF was measured 30 min before sacrifice.^30^

### 2.6 Behavioral assessments

Behavioral tests were performed 1 day before the PT insult, and then on day 1 and 7 post‐PT stroke. These tests, as described in our previous studies^21^ were the following.

- *Cylinder test*. The cylinder test was used to evaluate the asymmetry of paw usage in the PT stroke model. To perform the test, the rats were placed in a transparent glass cylinder (10 cm in diameter, 15 cm in height) for 2 min, during which the researcher counted the instances in which the animal made contact with the side of the cylinder using its left or right paws, or both paws simultaneously. We calculated the relative contralateral paw use using the following formula: score = (contralateral paw use/total paw use) * 100.
- *Adhesive removal test*. The adhesive removal test is a well‐established method for measuring somatosensory deficits in animals. We placed a small rectangular adhesive strip (0.35 × 0.45 cm) on the inner portion of each front paw of the rats and recorded the time it took for them to contact and remove the tape, with an upper time limit of 2 min.
- *Hanging wire test*. This test assesses multiple aspects of locomotor ability, including grip strength, endurance, and bodily coordination. Rats are placed on a wire hanging 60 cm above the ground. The time the animals spend on the wire (latency before falling), which reflects muscle strength, is recorded. The rat performance is rated on a scale of 0–4 based on different hanging behavior. A score of 0 is given if the animal falls immediately, while a score of 1 is given if the animal hangs onto the wire using both forelimbs. A score of 2 is given if the animal hangs on the wire using both forelimbs and attempts to climb onto the wire, while a score of 3 is given if the animal grasps the wire with both forelimbs as well as one or both hind limbs while attempting to climb onto the wire. Finally, a score of 4 is given if the animal grasps the wire with all four paws and wraps its tail around the wire. Each animal undergoes three trials during the testing session, and the highest score from three successive trials is taken as the final score for each animal.
- *Edge beam test*. To evaluate the motor coordination of rats, an Edge beam test was conducted. The rats were first habituated to a balance beam that was 100 cm in length and 7 cm in width and was elevated 100 cm above the horizontal surface of the ground, the day before testing. During the habituation phase, the rat home cage was placed at one end of the beam, and the rats were placed at three points of increasing distance from the cage, allowing them to cross the beam to return to their cage. During testing, the rats were placed at the far end of the beam, and their ability to traverse the beam was evaluated. The time taken to initiate movement (latency), the time taken to cross the beam (completion time), and the number of foot slips were measured. Latency was defined as the time taken to advance 20 cm beyond the start point, and completion time was defined as the interval between completion and the latency time point. Each trial had an upper time limit of 5 min to ensure that the test was not excessively stressful for the rats. The results of the Edge beam test provide insight into an animal's motor coordination, balance, and grip strength.
- *Ladder dexterity test*. The test was widely used to evaluate motor coordination. The apparatus used in this test consists of two Plexiglas side walls that are each 100 cm long, and they are linked by metal rungs that are 3 mm in diameter. The ladder rungs are irregularly spaced between 1 and 3 cm apart and are positioned 30 cm above the ground. During the test, the animals are placed at one end of the ladder and are allowed to cross the ladder to reach their home cages at the other end. The number of missteps made by the animals during the test is recorded and compared between different groups.

### 2.7 Brain perfusion and tissue preparation

As described in our previous study,^9^ the animals were subjected to transcardial perfusion with ice‐cold PBS while under deep anesthesia by isoflurane. Following this, the rat brains were transected around the infarct area, with half of them being utilized for immunofluorescence staining and the other half for ELISA and Western blot detection. For the brain tissues designated for immunofluorescence staining, they were post‐fixed overnight using 4% paraformaldehyde (PFA) and cryoprotected with a 30% sucrose solution. Subsequently, we obtained coronal sections (25 μm in thickness) from the ischemia‐infarcted cortex using a Cryostat (Leica RM2155; Nussloch, Germany).

### 2.8 Fluorescein isothiocyanate‐dextran (FITC‐dextran) and Evans blue injections

To measure microvascular perfusion, we administered FITC‐dextran (2000 kD, 50 mg/mL in 0.1 mL, 52,471, Sigma‐Aldrich, USA) via the tail vein 1 min prior to sacrifice. The brain was rapidly removed and fixed in 4% PFA at 4°C for 24 h, without a saline flush. To evaluate blood‐brain barrier permeability, we injected Evans blue (50 mg/kg, E2129; Sigma‐Aldrich, USA) intravenously through the tail vein and allowed it to circulate in the rats for 4 h.^9^ Following this, the rats underwent transcardial perfusion with ice‐cold PBS, followed by fixed with PFA. Brain sections were then cut and imaged to assess permeability.

### 2.9 Morphometric measurement of the brain vasculature

Morphometric determinations of changes in the vascular and astrocytic parameters, as well as vessel‐associated proteins, were performed on the peri‐infarct zone 7 days post‐stroke. Vascular structures were stained and visualized with an endothelial marker (Reca1). Vascular density (% area, vasculature area/total selection area) and vessel diameter were analyzed using Fiji software. Vascular length density was calculated as the ratio of the total vascular length divided by the total area (% area). In addition, Z‐stacked images of the peri‐infarct regions were acquired using a Zeiss AxioObserver with ApoTome. Representative 3D images were reconstructed, followed by surface rendering using Imaris 9.5.0 software (Bitplane, Switzerland). At least 3–5 cortical sections per animal were selected for staining and analysis, and the representative images are shown.

### 2.10 Automated Western immunoblotting

The JessTM Simple Western system (ProteinSimple, San Jose, CA, USA) is an automated capillary‐based size separation and nano‐immunoassay system. Rats were anesthetized and decapitated at 7 days post‐stroke, and the peri‐infarct area directly was dissected and rapidly frozen in liquid nitrogen. The frozen brain tissue and cell samples were homogenized in Tissue Extraction Reagent (FNN0071; Invitrogen, USA) with protease and phosphatase inhibitors (78,420, A32955; Thermo Scientific™, USA). The protein concentration of the lysates was quantified using a BCA Protein Assay Kit (23,227, Thermo Scientific Pierce Biotechnology, San Jose, USA). Capillary Western analysis was performed using the Simple Western System (ProteinSimple, USA) according to the instructions provided. Individual assays were performed for ZO‐1 (1:100, WA317222, Invitrogen, USA), Claudin 5 (1:20, 34–1600, Life Technologies, USA).

### 2.11 Isolation of cerebral vessels

After euthanasia, the meninges were removed and cortices were dissected in cold Dulbecco's phosphate‐buffered saline (02–0119‐0500; VWR life science, USA). Cortices were homogenized using a Dounce grinder and centrifuged (4°C) for 5 min at 2000 *g*. The vessel pellet was resuspended in 15% 70‐kDa dextran (31,390, Sigma‐Aldrich, USA) solution and centrifuged (4°C) for 15 min at 10,000 *g*. The upper layer, containing myelin and brain parenchymal cells, was removed. Vessels were retrieved by pipetting and transferred to a 40‐μm cell strainer. After being washed with cold DPBS (14,190,144; Gibco, USA), the vessels were collected in a tube by inverting the filter and adding 10 mL of MCDB 131 medium (10372–019; Gibco, USA) containing 0.5% fatty acid‐ and protease‐free BSA (16,609; Sigma‐Aldrich, USA). The suspension can be centrifuged (4°C) for 1dain at 5000 *g* to pellet the vessels for further study.^31^

### 2.12 Testosterone concentration measurement

The concentration of testosterone in serum and brain tissue samples and cellular samples was measured using an Elisa kit (ADI‐900‐065; Enzo, USA). In brief, 100 μL or 100 μg of the sample was added to a Goat anti‐mouse IgG microtiter plate. Subsequently, 50 μL of anti‐Testosterone antibody was added. The plate was then incubated at room temperature with a shaking speed of 500 rpm for 1 h. Then, 50 μL antibody conjugate was added followed by incubation for 1 h. After washing three times with 400 μL of wash buffer, 200 μL of pNpp substrate was added to each well and incubated for 1 h at 37°C without shaking. Afterward, 50 μL of stop solution was added into each well and the optical density was read at 405 nm. The testosterone level in each sample was calculated according to a standard curve and expressed as a percentage change vs. the Control group.

### 2.13 bEnd.3 cell culture and oxygen‐glucose deprivation (OGD) exposure

The mouse brain endothelial cell line, bEnd.3 was cultured in Dulbecco's Modified Eagle Medium/F12 (DMEM/F12, 11,320,033; Gibco, USA) supplemented with 10% fetal bovine serum (FBS, 6000044; Gibco, USA) and 1% Penicillin‐Streptomycin (15,070,063; Gibco, USA). The medium was refreshed every other day. Cells were divided and seeded at a density of 5 × 10^4^ cells/cm^2^ in charcoal‐dextran‐stripped fetal bovine serum in a 6‐well plate or 24‐well plate 24 h before the experiment. Sterile glass slides were placed in 24‐well cell culture plates. bEnd.3 cells were rinsed twice with PBS, and the medium was replaced with either glucose‐free and phenol red‐free DMEM (A1443001; Gibco, USA), or phenol red‐free DMEM/F12 (21,041,025; Gibco, USA) supplemented with glucose for OGD or control treatments, respectively. Cell plates were placed in a hypoxia chamber (Billups‐Rothenberg Inc., USA), and the air was replaced with OGD gas (95% N_2_ and 5% CO_2_) by flushing. Cells were exposed to OGD conditions for 18 h at 37°C.^32^ For the mechanism of testosterone studies, bEnd.3 cells were treated with 17β‐HSD5/AKR1C3 inhibitor (123,855, Sigma‐Aldrich, USA) (10 μM)^25^ trilostane (SML0141, Sigma‐Aldrich, USA) (25 μM),^33^ or flutamide (20 μM) for 1 h prior to OGD stimulation. We selected flutamide doses based on dose‐cytotoxicity experiments.

### 2.14 Immunofluorescence staining and microscopy

Immunofluorescence staining was performed as described in a previous study.^28^ Briefly, after brain slice preparation, 3–5 sections from each animal were selected for microscopy imaging. The coronal slices collected were permeabilized with 0.4% Triton X‐100 for 8 h and blocked with 10% normal donkey serum for 1 h at room temperature. Next, the brain sections were incubated overnight with the appropriate primary antibody at room temperature. The following primary antibodies were used: anti‐testosterone (1:2, ADI‐900‐065; Enzo Life Sciences, USA), CD31 (1: 300, AF3628; R&D system, USA), Reca1 (1:300, 14–0360‐82; Invitrogen, USA), 3β‐HSD1 (1:200, sc‐515,120; Santa Cruz Biotechnology, USA), 17β‐HSD5 (1:300, MAB7679; R&D System, USA), ZO‐1 (1:300, 40–2200; Invitrogen, USA), Claudin 5 (1:200, 34‐1600; Life Technologies, USA), cle‐Caspase 3 (1:300, #9661; Cell Signaling Technology, USA), GFAP (1:500, ab7260; Abcam, USA), Iba1 (1:400, 019‐19,741; FUJIFILM Wako Pure Chemical Corporation, Japan), MAP2 (1:400, 17,490‐1‐AP, Proteintech Group, USA). The following day, brain slices were washed three times with 0.1% Triton X‐100 followed by incubation with Alexa Fluor‐labeled donkey anti‐mouse/goat secondary antibodies (555/647/488; Thermo Fisher Scientific) for 1 h at room temperature. After washing, the sections were mounted with DAPI Fluoromount‐G® Mounting Medium (SouthernBiotech, Birmingham, AL, USA).

Cells were rinsed with PBS and then fixed with 4% PFA for 15 min. Subsequently, they were permeabilized for 10 min using 0.4% Triton X‐100 in PBS. Afterward, the cells were incubated with the primary antibody overnight at 4°C. Following three washes with PBS, the secondary antibody was added, and the cells were incubated for 1 h at room temperature. Finally, the nuclei were stained with DAPI and mounted.

All fluorescent images were captured by Zeiss AxioObserver with ApoTome (Carl Zeiss, USA) using a 40×or 100×oil immersion objective and analyzed using ImageJ software (Version 1.49; NIH, Bethesda, MD, USA) by investigators blinded to the experimental groups. 3β‐HSD1 and 17β‐HSD5 were measured by 3D quantification within selected vessels from the 3D contour surface. Z‐stack images were captured at 1 μm z step distance apart. All imaging was carried out using the same exposure time and emission gain for all spinal slices.

### 2.15 TUNEL staining

The apoptotic cells in the cerebral vessels and bEnd.3 cells were detected using a Click‐iT® Plus TUNEL assay kit (Thermo Fisher Scientific, USA) according to the manufacturer's protocol, as described in our previous study.^9^ Cell counts in the examined sections were then averaged to provide a single value for each specific group.

### 2.16 Statistical analysis

During data acquisition and analysis, the investigator was blinded to treatment group assignment. All data were tested for normality using the Shapiro‐Wilk test (appropriate for less 50 sample sizes) and all error bars are presented as standard deviation of mean (SD) in bar graphs. All analyses were performed using GraphPad Prism (GraphPad, USA) and SPSS software (IBM, USA). Kruskal‐Wallis test (a nonparametric alternative to one‐way ANOVA) was used for data which did not pass the test for normality. Statistical comparisons between the two groups were performed using unpaired *t*‐tests. Comparisons between multiple groups were made using one‐way or two‐way ANOVA, using Bonferroni's *post hoc* test for comparisons between all groups. A level of *p* < 0.05 was considered statistically significant.

## 3 RESULTS

### 3.1 PBMT increased the testosterone concentration *in vitro* and *in vivo* stroke models

The experimental timeline of the study is illustrated in Figure 1A. As shown in Figure 1B–D, the testosterone concentration following PT stroke insult was significantly lower in the brain microvessel samples, but not in the serum or total protein samples of the cortical peri‐infarct area. PBMT increased the testosterone concentration in the brain vessels and infarct area of PT‐stroke rats. Surprisingly, we detected no change in the serum testosterone concentration (Figure 1B). Immunofluorescence results showed that PT stroke decreased the vessel‐associated testosterone level in the infarct area, while PBMT reversed this change (Figure 1F,G). To further test our hypothesis that PBMT could provide vascular protection via testosterone, we performed a series of cellular experiments using bEnd.3 cells. As displayed in Figure 1I,J, ELISA and immunofluorescence assay results revealed that OGD exposure decreased the expression levels of testosterone in bEnd.3 cells. These effects were significantly reversed following PBMT.

### 3.2 Flutamide blocked PBMT‐induced improvement in BBB permeabilization, vascular morphometric parameters, and CBF after PT‐stroke in rats

**FIGURE 2:**
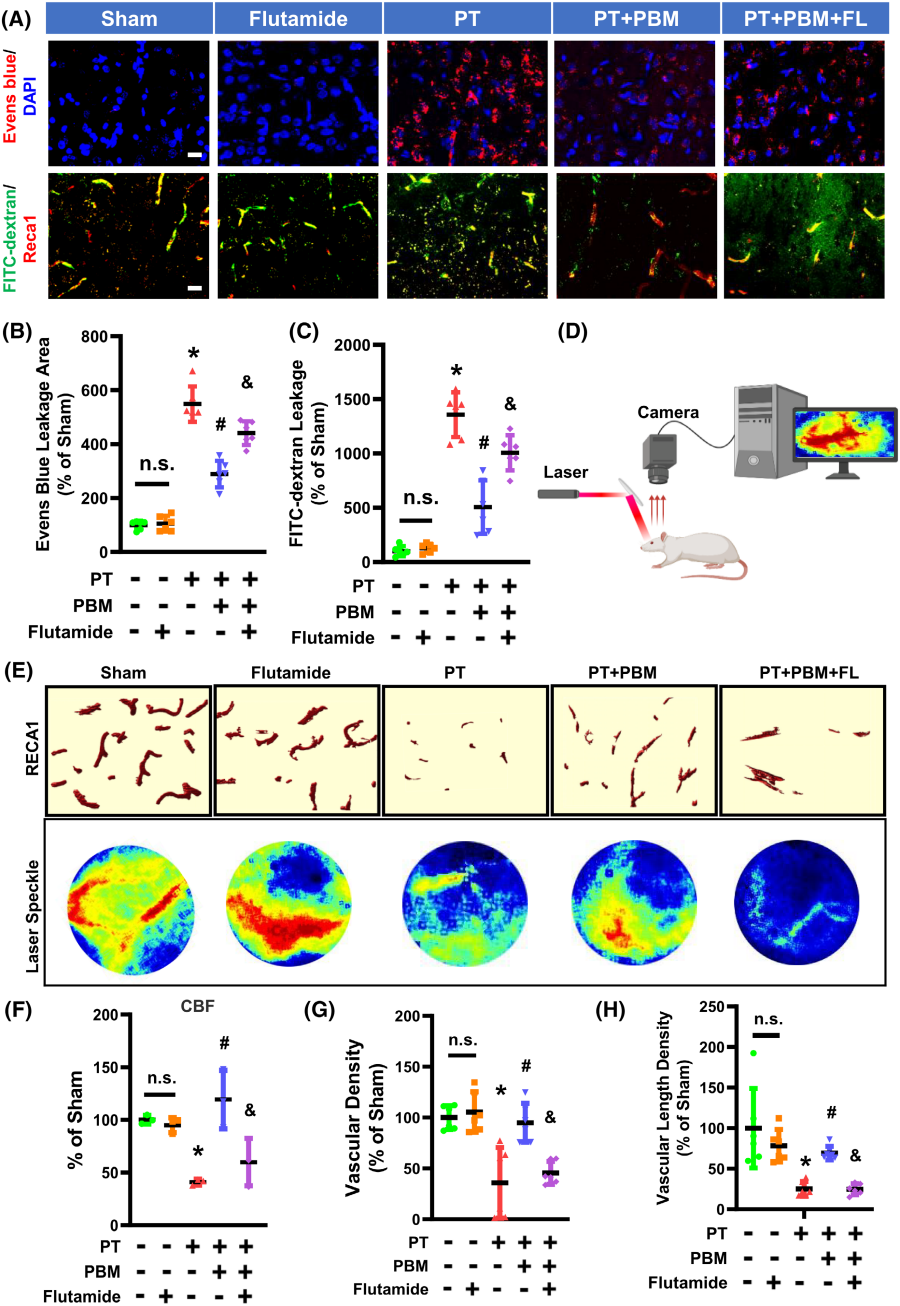
Flutamide inhibits the improvement in blood‐brain barrier (BBB) permeability, vascular morphometric parameters, and cerebral blood flow (CBF) induced by PBMT in the peri‐infarct region of PT‐stroke rats. (A) Representative fluorescence images of Evans blue (red), FITC‐dextran (green), and Reca1 (red) in the peri‐infarct zone. Nuclei were counterstained with DAPI (blue). (B, C) Quantitative analysis of extravascular Evans blue and FITC fluorescence intensity was performed using ImageJ software. (D) A schematic of the laser speckle contrast imaging system. (E) Representative 3D reconstruction (Bitplane Imaris software) of Reca1 staining and flux images of cortical vasculature in the peri‐infarct regions are shown. (F) Changes in CBF were calculated as percentage changes relative to Sham group. (G) Quantitative analysis of vascular density was shown as percentage change relative to Sham group. (H) Quantitative analysis of vascular length density was performed as percentage change relative to Sham. All data are expressed as mean ± SD (*n* = 5–6). One‐way ANOVA followed by post hoc Bonferroni's test was used for analysis. * indicates *p* < 0.05 vs. Sham group; # indicates *p* < 0.05 vs. PT‐stroke group; & indicates *p* < 0.05 vs. PT + PBM group. “n.s.” indicates no significant difference (*p* > 0.05). FL, flutamide. Scale bar = 20 μm.

To further investigate the effect of vascular testosterone on vascular permeability and function, we assessed the leakage area of Evans blue and FITC‐dextran in response to flutamide (testosterone receptor antagonist) and PBMT stimulation of PT‐stroke rats. Representative microscopy images of FITC‐dextran and Evans blue extravasation in the peri‐infarct region are shown in Figure 2A. Considerable leakage of Evans blue and FITC‐labeled dextran was observed in the cortical peri‐infarct area of PT rats, but was reversed in PBMT group rats (Figure 2B,C). When stimulated with flutamide, FITC‐dextran and Evans blue leakage was increased dramatically in PT‐stroke rats.

The CBF was examined by laser speckle imaging (Figure 2D), and the results showed that rats subjected to PT stroke showed a significant decrease in CBF compared with control rats, while PBMT rats had a significantly higher CBF (Figure 2E,F). This effect was inhibited when combined with flutamide treatment. Finally, vascular morphology was similarly impaired in animals receiving PT stroke as shown by vascular density and vascular length (Figure 2G–I). These altered morphological metrics were significantly reversed by PBMT. However, these changes were abolished in PBMT rats that received *icv* injection of flutamide. These data demonstrate blockading androgen receptor activity with flutamide effectively inhibited PBMT‐associated improvements in BBB permeabilization, vascular morphology, and CBF.

### 3.3 PBMT promoted the expression of TJ proteins both *in vivo* and *in vitro* in a flutamide‐dependent manner

**FIGURE 3:**
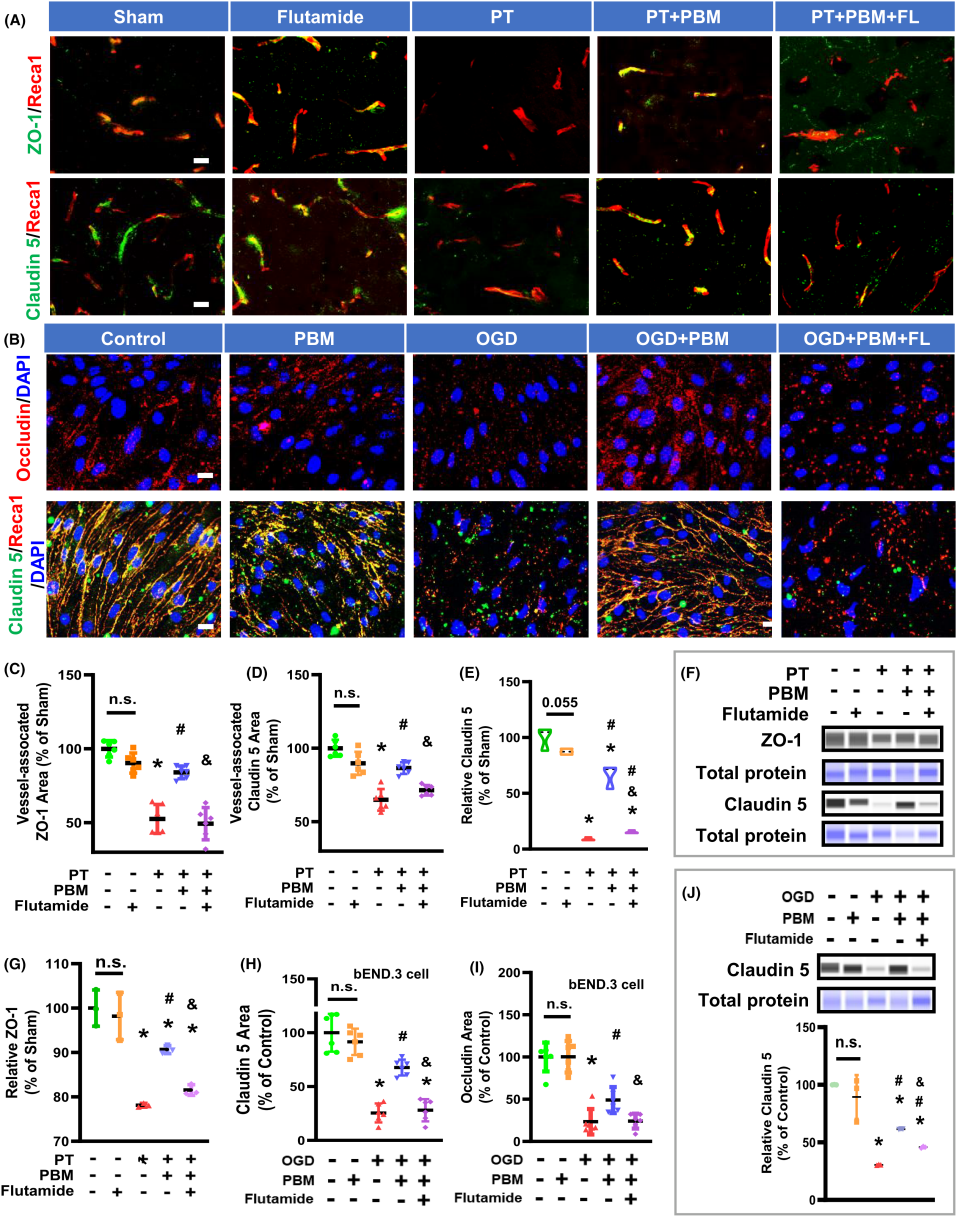
Flutamide reduces PBMT‐mediated upregulation of TJ proteins in PT‐stroke rats and OGD‐treated bEND.3 cells. (A) Representative fluorescence images of ZO‐1 (green), Claudin 5 (green), and Reca1 (red) in the peri‐infarct areas. (B) Representative immunofluorescence images for Occludin (red), Claudin 5 (green), and Reca1 (red) in bEND.3 cells. Nuclei were counterstained with DAPI (blue). The data are expressed as mean ± SD (*n* = 5–6). (C, D) Quantitative analysis of vessel‐associated ZO‐1 and Claudin 5 were performed using ImageJ software. (E–G) Western blotting and quantitative analysis of the ZO‐1 and Claudin 5 levels using protein samples from the peri‐infarct brain region. The data are expressed as mean ± SD (*n* = 3). (H, I) Quantitative analysis of Claudin 5 and Occludin immuno‐intensity in bEND.3 cells. The data are expressed as mean ± SD (*n* = 5–6). (J) Western blotting and quantitative analysis of the Claudin 5 proteins in bEND.3 cells. The data are expressed as mean ± SD (*n* = 3). One‐way ANOVA followed by *post hoc* Bonferroni's test was used for analysis. * indicates *p* < 0.05 vs. Sham group or Control group; # indicates *p* < 0.05 vs. PT‐stroke group or the OGD group; & indicates *p* < 0.05 vs. PT + PBM group or OGD + PBM group. “n.s.” indicates no significant difference (*p* > 0.05). OGD, Oxygen‐glucose deprivation. Scale bar for tissue = 20 μm. Scale bar for cells = 10 μm.

The TJ proteins ZO‐1, Claudin 5, and Occludin mediate endothelial adherens junctions, regulate cell‐cell tension, contribute to angiogenesis, and participate in BBB formation.^34^, ^35^ Loss of Claudin 5 and ZO‐1 results in increased vascular permeability. To assess whether PBMT induced an increase in the expression of TJ proteins, we subjected PT stroke rats to 7 days of PBMT. The Western blotting and immunofluorescence results demonstrated that the vessel‐associated TJ proteins ZO‐1 and Claudin 5 were downregulated after a PT stroke (Figure 3A,C–G). However, PBMT upregulated these proteins. Next, we evaluated the biological effects of testosterone by treating rats with the testosterone receptor agonist flutamide. Results showed that Claudin 5 and ZO‐1 expression was significantly decreased when combined with flutamide treatment (Figure 3A,C–G).

The MTT assay indicated that flutamide did not affect cellular viability at a concentration of 20 μM or lower for 48 h (Figure S1A,B). Therefore, we examined the expression of TJ proteins in the presence of 20 μM flutamide. We observed higher protein expression of Claudin 5 and increased immunofluorescence intensity of Claudin 5 and Occludin after PBMT in bEnd.3 cells that underwent OGD, compared to those treated with OGD alone. However, these changes were reversed by flutamide (Figure 3B, H‐J).

### 3.4 Flutamide abrogated the inhibitory effect of PBMT on cerebrovascular endothelial cell apoptosis both *in vivo* and *in vitro*

**FIGURE 4:**
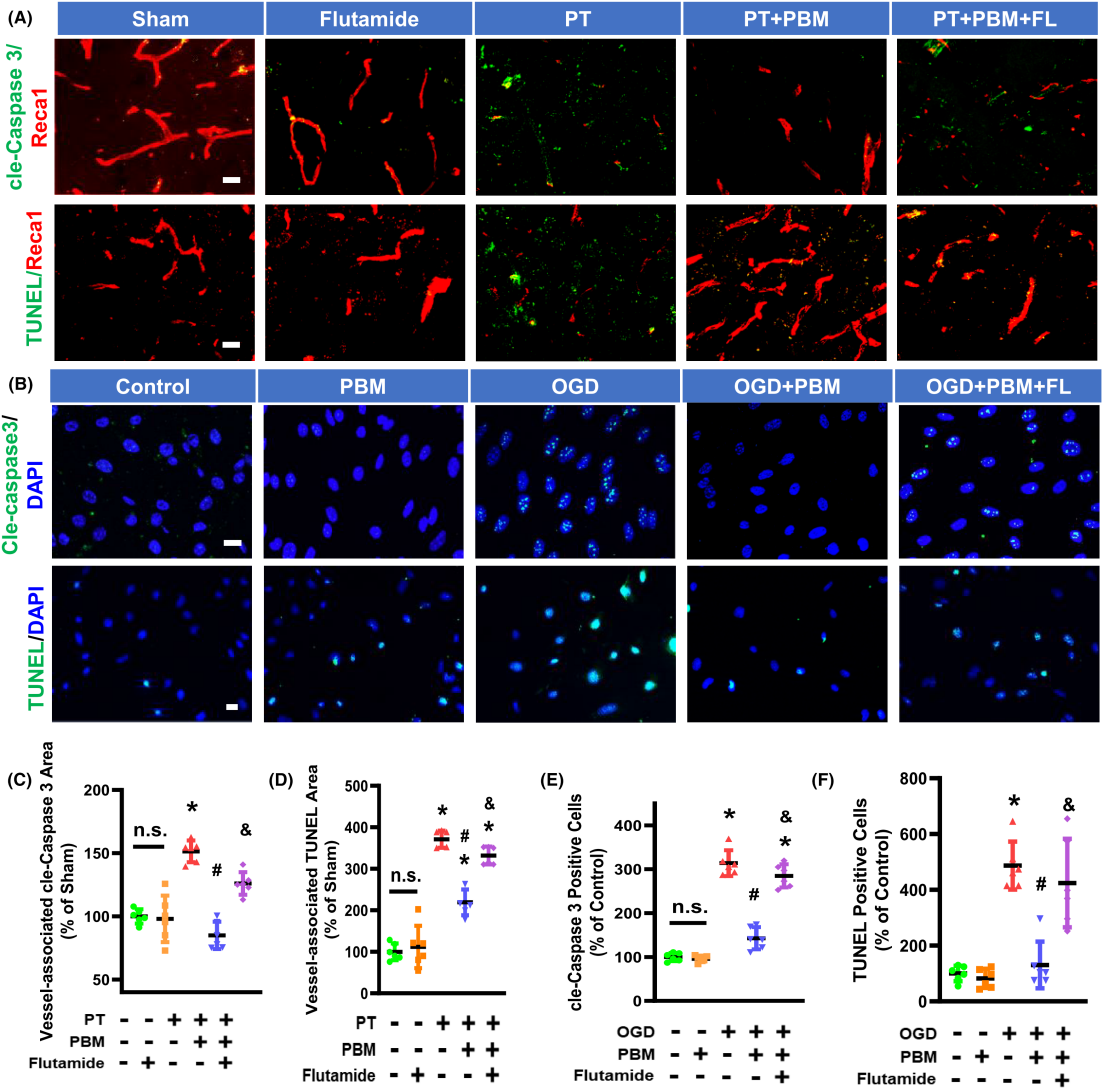
Flutamide reverses PBMT‐mediated inhibition of vessel‐associated cle‐Caspase 3 and TUNEL in PT‐stroke and OGD‐treated bEND.3 cells. (A) Representative immunofluorescence images of cle‐Caspase 3 (green) and Reca1 (red), and TUNEL staining (green) in the peri‐infarct brain areas. (B) Representative fluorescence images for cle‐Caspase 3 (green), TUNEL (green), and DAPI (blue). (C, D) Quantitative analysis of vessel‐associated cle‐Caspase 3 and TUNEL were performed using ImageJ software (*n* = 5–6 per group). (E, F) Quantitative analysis of the percentage of cell with cle‐Caspase 3‐positive nuclei and TUNEL‐positive nuclei were performed using ImageJ software in bEND.3 cells (*n* = 5–6). All data are expressed as mean ± SD. One‐way ANOVA followed by *post hoc* Bonferroni's test was used for analysis. * indicates *p* < 0.05 vs. Sham or Control group; # indicates *p* < 0.05 vs. PT‐stroke or OGD group; & indicates *p* < 0.05 vs. PT + PBM or OGD + PBM group. “n.s.” indicates no significant difference (*p* > 0.05). FL, flutamide; OGD, Oxygen‐glucose deprivation. Scale bar for tissue = 20 μm. Scale bar for cells = 10 μm.

Subsequently, we investigated the occurrence of apoptosis in the brain vasculature following PBMT and PT stroke intervention by staining for the endothelial marker Reca1 and TUNEL for apoptosis. The colocalization of these two signals indicates apoptotic vascular endothelial cells. As shown in Figure 4A,C,D, the levels of vessel‐associated apoptotic proteins (Reca1^+^/cle‐Caspase 3^+^) and apoptotic endothelial cells (Reca1^+^/TUNEL^+^) were significantly higher in PT group than Sham group. PBMT application effectively reduced apoptosis. We then investigated whether flutamide could abrogate the effect of PBMT on endothelial cell apoptosis. The results showed that flutamide increased both TUNEL and cle‐Caspase 3‐labeled cells in stroke rats receiving PBMT.

In our *in vitro* study using OGD‐exposed bEnd.3 cells, as displayed in Figure 4B,E–F, the numbers of cle‐Caspase 3 and TUNEL‐labeled cells were significantly reduced by PBMT. However, adding flutamide remarkably suppressed PBMT effects on Caspase 3 activation and apoptosis, suggesting an effect mediated by the testosterone receptor.

### 3.5 PBMT‐alleviated behavioral deficits in stroke rats are partly mediated via AR

**FIGURE 5:**
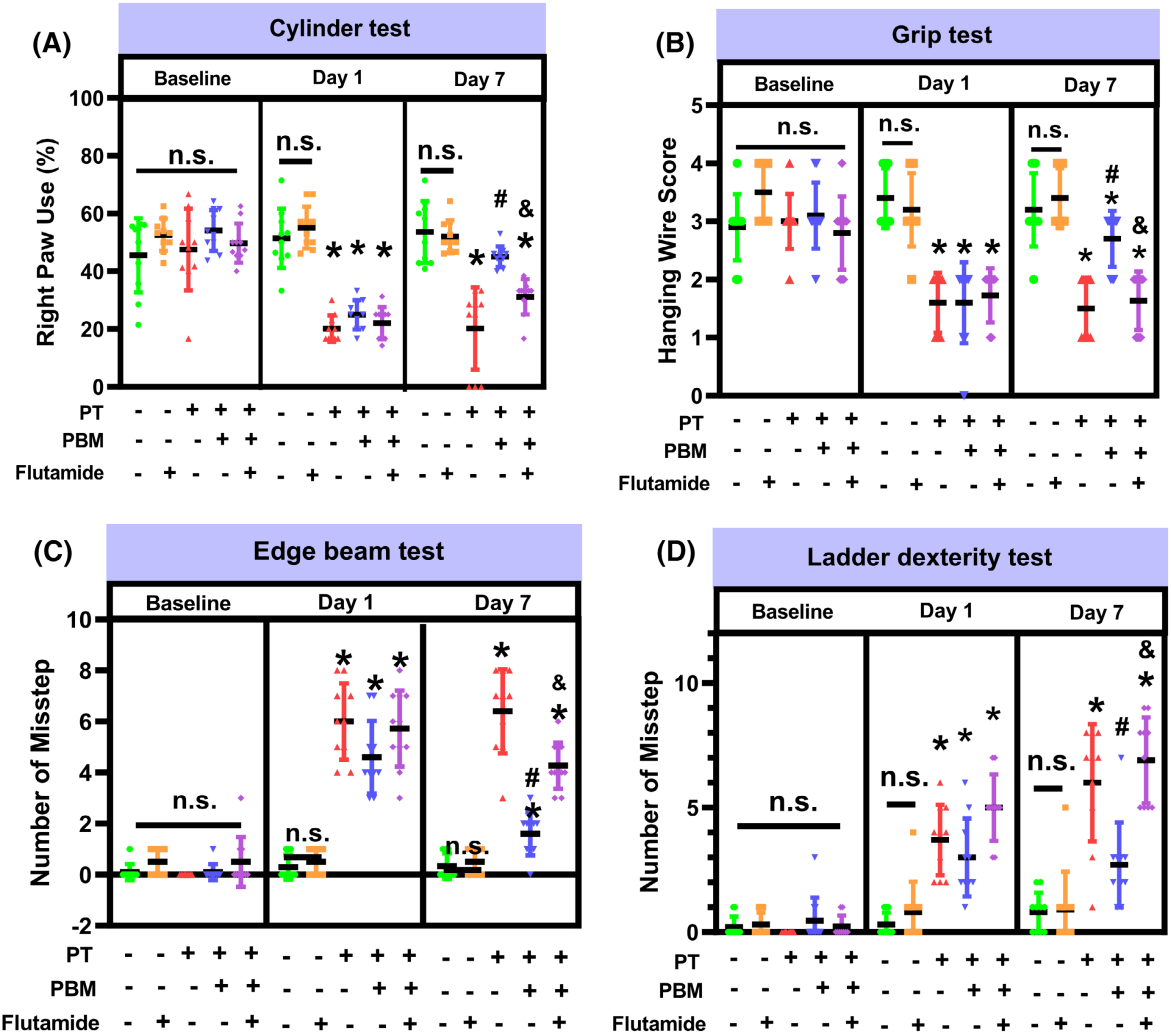
Flutamide attenuates PBMT‐alleviated behavioral deficits in PT‐stroke rats. (A) The cylinder test measured forelimb use. (B) The grip test analyzed forelimb motor coordination and grip strength. (C) The edge beam test was used to assess motor balance and coordination. (D) The number of missteps of the ladder dexterity test were performed to investigate motor coordination. All data are expressed as mean ± SD (*n* = 10). One‐way ANOVA followed by *post hoc* Bonferroni's test was used for analysis. * indicates *p* < 0.05 vs. Sham group; # indicates *p* < 0.05 vs. PT‐stroke group; & indicates *p* < 0.05 vs. PT + PBM group. “n.s.” indicates no significant difference (*p* > 0.05).

PT‐stroke rats showed reduced use of the impaired contralateral (right) forelimb in the cylinder test (Figure 5A), lowered wire scores (Figure 5B). In addition, rats in the PT group made more missteps in the edge beam test (Figure 5C) and ladder dexterity test (Figure 5D), and spent more time removing the adhesive tape (Figure S2A) compared with Sham group. These behavioral deficits were significantly improved by PBMT, with the greatest effect observed on day 7. To gain insights into whether PBMT‐induced testosterone was responsible for the beneficial effects of PBMT, the effect of the selective androgen receptor antagonist flutamide on PBMT‐improved behavior was further assessed. As depicted in the baseline measures in Figure S2B, normal rats receiving an *icv* infusion of flutamide showed no significant difference in the behavioral results. However, PBMT‐rats receiving flutamide displayed a significant reduction in the use of the impaired contralateral forelimb (Figure 5A) and wire scores (Figure 5B) compared to rats receiving PBMT alone. Similarly, PBMT rats administered flutamide had a marked increase in missteps in the edge beam (Figure 5C) and ladder dexterity tests (Figure 5D), and spent a longer duration of time removing adhesive tape (Figure S2A) compared to PBMT‐group rats. However, the completion time spent in the ladder dexterity text was not significantly different between groups (Figure S2B). These findings suggest that the positive effects of PBMT on behavioral improvement were mediated, at least in part, by PBMT‐induced testosterone production in the brain.

### 3.6 Expression pattern of testosterone‐synthesis enzymes, 3β‐HSD1 and 17β‐HSD5, *in vivo* and *in vitro*, and the effects of PBMT

**FIGURE 6:**
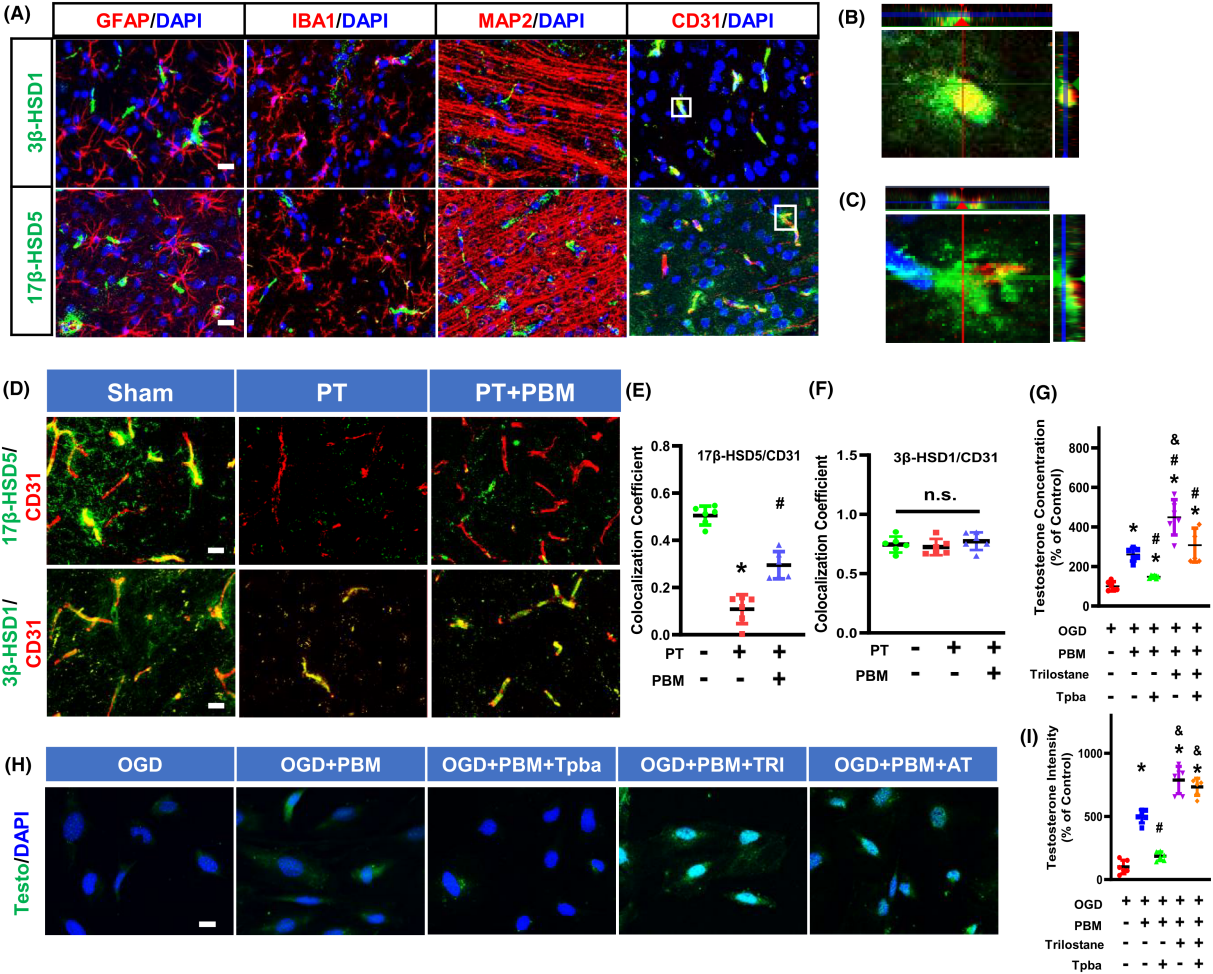
Expression of 3β‐HSD1 and 17β‐HSD5 in the rat brain, and the effects of 3β‐HSD1/17β‐HSD5 inhibitors on PBMT‐induced testosterone production. (A) Representative immunofluorescence staining images of 17β‐HSD5 (green), 3β‐HSD1 (green), GFAP, IBA1, MAP2, and CD31 in normal animal brains. Nuclei were counterstained with DAPI. (B) Orthogonal images of 3β‐HSD1 (green)/CD31 (red) are represented in the x‐z and y‐z directions. (C) Orthogonal images of 17β‐HSD5 (green)/CD31 (red) are represented in the x‐z and y‐z directions. (D) Representative immunofluorescence staining images of 3β‐HSD1 (green), 17β‐HSD5 (green) and CD31 (red) in the peri‐infarct area. (E, F) Quantitative analysis of colocalization of 3β‐HSD1 or 17β‐HSD5 with CD31 in peri‐infarct area of rat brain (*n* = 5–6). (G) Testosterone concentration was measured by ELISA after treatment with 25 μM Trilostane or 10 μM Tpba. (H) Representative immunofluorescence staining images of Testosterone (testo) and DAPI after treatment with Trilostane or Tpba. (I) Quantitative analysis of testosterone immunointensity in bEND.3 cells. All data are expressed as mean ± SD (*n* = 6). One‐way ANOVA followed by post hoc Bonferroni's test was used for analysis. Scale bar for tissue = 20 μm. Scale bar for cells = 10 μm. Testo, Testosterone; AT, Tpba and Trilostane; TRI, Trilostane; OGD, Oxygen‐glucose deprivation. * indicates *p* < 0.05 vs. Sham or Control group; # indicates *p* < 0.05 vs. PT stroke group or OGD group; & indicates *p* < 0.05 vs. PT + PBM group or OGD + PBM group or OGD + PBM + AI group. “n.s.” indicates no significant difference (*p* > 0.05).

Testosterone can be synthesized by the enzymes 3β‐HSD1 and 17β‐HSD5 in the brain. As shown in Figure 6A–C, the protein expression of 3β‐HSD1 and 17β‐HSD5 were not colocalized with MAP2 (neuronal marker), Iba1 (microglia), or GFAP (astrocytes), but were well colocalized with CD31 (endothelial cells). These results suggest that 3β‐HSD1 and 17β‐HSD5 are mainly expressed in cerebrovascular vessels in normal rats. As shown in Figure 6D–F, PT‐stroke animals displayed a significantly decreased endothelial 17β‐HSD5, but not 3β‐HSD1 in the peri‐infarct brain regions. In addition, PBMT could increase levels of 17β‐HSD5 but not 3β‐HSD1 in the cerebrovasculature.

After a 72‐h intervention with 3β‐HSD1 inhibitor (trilostane) and 17β‐HSD5 inhibitor [3‐(4‐(Trifluoromethyl)phenylamino)benzoic acid, Tpba], bEnd.3 cells did not experience significant cellular viability changes (Figure S1C), suggesting that these inhibitors had no toxic effects. However, ELISA and immunofluorescence results showed that the PBMT‐induced up‐regulation of testosterone concentration was blocked by a 17β‐HSD5 inhibitor in bEnd.3 cells. The use of 3β‐HSD inhibitor, in contrast, had no dampening effect on testosterone upregulation (Figure 6G–I). Taken together, these results suggest that PBMT may target the key testosterone‐biosynthesis enzyme 17β‐HSD5 to induce its positive effects following stroke.

## 4 DISCUSSION

The present study is the first to demonstrate a reduction in cerebrovascular testosterone 7 days after a PT stroke in rats. Additionally, we found that PBMT increased the cerebrovascular testosterone concentration both *in vitro* and *in vivo*. Blocking the biological effects of testosterone using a testosterone receptor antagonist inhibited the increase in CBF and TJ protein expression, as well as affecting the vascular morphology and behavioral improvements induced by PBMT in PT stroke rats. This suggests that PBMT can provide vascular protection and can ameliorate behavioral deficits partly by an increase in cerebrovascular testosterone after a stroke insult. Cellular experiments further confirmed that the effect of PBMT on testosterone was partly mediated by 17β‐HSD5 in cerebrovascular endothelial cells.

Testosterone is known to promote vascular remodeling,^36^ and can modulate endothelial function.^37^ However, it was unclear whether there is a direct link between stroke and cerebrovascular testosterone. Previous studies have reported a decrease in serum testosterone in the immediate post‐stroke period, which tends to normalize over time especially in men.^5^, ^38^ Our data showed a decrease in the cerebrovascular testosterone level 7 days after PT stroke, while there was no significant change in testosterone in the serum or the peri‐infarct cortex. Previous research has indicated a significant decrease in both total and free testosterone levels following a stroke.^38^ This variance might stem from the stroke model employed. It is suggested that focal cerebral ischemia might not be sufficient to prompt a notable change in serum testosterone concentrations. Furthermore, discrepancies observed might also relate to the timing of tests. Clinical trials have noted a tendency for total and free testosterone levels to normalize approximately 6 months after a stroke.^38^ The shift in serum testosterone may lessen after a seven‐day recovery in our study. Local testosterone produced and concentrated in endothelial cells could be detected, as it is synthesized and concentrated in isolated cerebral vessels. However, we did not observe significant changes in total protein samples. This lack of detection may be attributed to the diluted nature of testosterone content in the total proteins. Presently, there was no research confirming the variations in testosterone content within the neurons and astrocytes following stroke. The potential changes in testosterone within neurons and astrocytes under ischemic conditions might differ from those observed in blood vessels. However, further exploration is necessary to ascertain the precise underlying reasons.

We also found that OGD decreased the testosterone concentration in cultured brain microvascular endothelial cells (bEnd.3 cells). The findings indicate that stroke development may be influenced by testosterone levels within the cerebral vessels, rather than testosterone levels in the serum. Cerebrovascular testosterone may be a highly promising biomarker for predicting the occurrence and outcomes of ischemic stroke.

The presence of enzymes necessary for androgen biosynthesis from cholesterol has been identified in the adult human brain,^39^ and it has been suggested that testosterone is synthesized de novo in the adult brain.^40^ Currently, there is a paucity of literature regarding local testosterone synthesis in the cerebral vessels. Our data showed that PBMT increased the vessel‐associated testosterone concentration in PT stroke rats and in OGD‐treated bEnd.3 cells. These results suggest the local cerebrovascular synthesis of testosterone that could be increased by PBMT. Previous studies have reported the effect of PBMT on testosterone levels, demonstrating that PBMT applied to the testes increased serum testosterone levels in rats^23^ and mice.^41^ However, in our study, we did not observe any significant change in serum testosterone levels after PBMT in PT stroke rats. This discrepancy may be due to differences in the location of irradiation (testes vs. brain). Our results suggest that the elevation of cerebrovascular testosterone alone would not be sufficient to alter serum testosterone levels.

Previous studies have shown that a stroke could lead to the deterioration of BBB integrity and impairment of vascular function in both the acute and subacute stages.^9^, ^42^ PBMT has been demonstrated to increase CBF and preserve endothelial cells in stroke‐damaged brain lesions.^21^, ^43^ Additionally, PBMT has demonstrated the ability to mitigate cerebrovascular leakage in models of Parkinson's disease.^44^ Our research aligns with these findings, illustrating that PBMT reversed the compromised CBF and increased BBB permeability observed in post‐stroke rats. The overall increase in BBB permeability in stroke has been largely associated with differences in the expression of TJ protein, such as ZO‐1 and Claudin 5.^45^ In agreement with this, our results showed that the ZO‐1 and Claudin 5 were decreased both in vitro and *in vivo* stroke models, and that PBMT could reverse these changes. Other studies also reported that PBMT increases the TJ proteins in retinal pigment epithelium cell.^46^ The mechanism of PBMT predominantly involves the stimulation of Cytochrome C oxidase within the mitochondrial respiratory chain. While ongoing research is delving into the molecular mechanisms, it was noted that PBMT activates multiple signaling pathways. For instance. In neurons, PBMT triggers the extracellular signal‐regulated kinase/cAMP‐responsive element‐binding signaling pathway.^47^ In diabetic fibroblast cells, PBMT activates the PI3K/AKT pathway,^48^ and it has been observed to promote neural stem cells via the AKT/GSK‐3β/β‐catenin pathway.^49^ There is a scarcity of research investigating PBMT's impact on cerebrovascular cells in stroke rats. Further exploration into the potential mechanisms underpinning PBMT's impact on vascular structure and function is poised to be a key focus of future research.

Additionally, there was a significant decrease in total vessel length, volume, and number of branches in stroke patients.^50^ Our study demonstrated that PBMT improved vascular morphology, including vascular density, vascular length and lumen diameter in PT stroke rats. It has been reported that photobiomodulation regulates cytokine release and angiogenesis during oral wound healing in rats.^51^ However, there is limited research on the impact of PBM on cerebrovascular morphology.

As mentioned above, PBMT also increased ular testosterone concentration. Based on these results, we hypothesized that PBMT could improve vascular morphology and function via increasing the cerebrovascular testosterone concentration. To verify this conjecture, we used the testosterone receptor antagonist, nonsteroidal flutamide. As expected, flutamide inhibited PBMT‐induced beneficial effects on vascular morphology, CBF and BBB permeability along with TJ protein expression in PT stroke rats, indicating that testosterone may be involved in the beneficial effects of PBMT on vascular morphology and function.

In addition, previous studies have reported that cerebral ischemic/reperfusion (I/R) injury can lead to endothelial necroptosis *in vivo*,^52^ and OGD could trigger endothelial cell death by increasing cleaved‐Caspase 3 and TUNEL‐positive cells *in vitro*.^53^ This is consistent with our data showing increased levels of TUNEL and cleaved‐Caspase 3 in the vascular endothelium after PT stroke or OGD exposure. It has been suggested that PBMT could provide a vascular protective effect in peripheral tissues.^22^ Although our previous study demonstrated PBMT's ability to reduce caspase‐3 activity in neurons within the hypoxic and ischemic model,^54^ there is a scarcity of reports regarding the impact of PBMT on cerebrovascular cell apoptosis. Furthermore, our results revealed that PBMT reduced the number of cleaved‐Caspase 3 and TUNEL‐positive endothelial cells in both bEnd.3 cell line and in rat brain, providing evidence that PBMT exerts a protective effect on cerebral vessels by inhibiting endothelial cell apoptosis in both *in vitro* and *in vivo* stroke models. Testosterone is known to modulate vascular endothelial cell growth and proliferation.^55^, ^56^ But much less is known about the mechanisms of the vascular protective effects of testosterone after PBMT for cerebral ischemic stroke. Our observation that flutamide administration led to an increase in TUNEL and cle‐Caspase 3‐positive vascular endothelial cells in both PT‐stroke rats and cells treated with OGD, indicating that testosterone may be at least partly responsible for the vascular protective effects of PBMT.

PBMT has been reported to improve behavioral deficits in rat models of ischemic stroke.^19^ Our study confirmed that PT‐stroke rats exhibited behavioral deficits, and that PBMT reversed these changes. However, the role of testosterone in the mitigation of post‐stroke behavioral deficits induced by PBMT was unclear. Our results showed that blocking testosterone activity in the brain eliminated PBMT‐induced improvement in behavioral deficits in PT‐stroke rats. These findings provide direct evidence of the involvement of testosterone in the improvement of behavior induced by PBMT following ischemic stroke.

Subsequently, we conducted cellular experiments to investigate the mechanisms by which PBMT could increase vascular testosterone concentration. Testosterone synthesis involves several key enzymes and proteins, including 17α‐hydroxylase, 3β‐HSD, and 17β‐HSD.^24^ The final conversion of androstenedione to testosterone is catalyzed by 17β‐HSD.^57^ Nonetheless, the role of 17β‐HSD enzymes in different cells has not been well defined in the brain. It has been reported that 17β‐HSD5 is expressed in the endothelial cells of blood vessels within prostate tissue.^58^ Hence, we hypothesized that PBMT could increase the testosterone level via stimulation of 17β‐HSD5 in the cerebral vessels. In accordance with the hypothesis, our data showed that PBMT caused an increase in vessel‐associated 17β‐HSD5 levels, but not 3β‐HSD1 levels in PT‐stroke rats. The addition of a 17β‐HSD5 inhibitor, but not a 3β‐HSD1 inhibitor, resulted in a partial decrease in the testosterone synthesis promoted by PBMT in OGD‐treated bEnd.3 cells. The results of our study revealed that PBMT increased vascular testosterone, which could be partially mediated by 17β‐HSD5. The experiments should be repeated in female rats to see whether testosterone was also involved in the known improvement that PBMT can produce in female animals that had been subjected to a stroke.

In conclusion, the present study highlights the effect of stroke on cerebrovascular testosterone levels, and proposes that cerebrovascular testosterone could be a potential prognostic indicator in stroke. Our findings provide the first direct evidence that PBMT can increase vascular testosterone levels which may explain its protective effects on the cerebral vasculature. Given the potential risks associated with testosterone therapy, the ability of PBMT to increase vascular testosterone levels and improve post‐stroke behavioral deficits makes it a highly promising intervention without any side effects, for the treatment of cerebrovascular diseases including ischemic stroke. This experiment has certain limitations as we did not specifically knock out 17βHSD5 within brain vasculature, which is a direction we intend to pursue in our subsequent research. Additionally, our study did not investigate the effects of other androgens. There is existing research indicating that lower levels of dehydroepiandrosterone (DHEA) are associated with an increased risk of ischemic stroke, and DHEA shows promise in mitigating oxidative stress and inflammation post‐arterial injury.^59^, ^60^ Exploring whether PBMT has an effect on DHEA warrants further investigation. Our experiment not only demonstrates the feasibility of PBMT as a treatment strategy for cerebrovascular diseases but also suggests that cerebral vascular testosterone could serve as a significant target in stroke. Hence, further research on post‐stroke cerebral vascular testosterone could be a promising avenue for future investigations.

## AUTHOR CONTRIBUTIONS

Y.F., Q.Z., and M.R.H. designed research; Y.F., Z.H., X.M., and X.Z. performed research; C.Y.‐C.W., R.H.‐C.L., and H.W.L. contributed equipment; Y.F. and Q.Z. analyzed data; Y.F., M.R.H., and Q.Z. wrote the article.

## FUNDING INFORMATION

Research reported in this publication was supported by the National Institute on Aging of the National Institutes of Health under Award Number RF1AG058603. The content is solely the responsibility of the authors and does not necessarily represent the official views of the National Institutes of Health. MRH was supported by US NIH Grants R01AI050875 and R21AI121700.

## CONFLICT OF INTEREST STATEMENT

The authors declare that they have no known competing financial interests or personal relationships that could have appeared to influence the work reported in this article.

## Supporting information


Figure S1.

## Data Availability

The data that support the findings of this study are available from the corresponding author upon reasonable request.
