# Correction to “Activation of testosterone‐androgen receptor mediates cerebrovascular protection by photobiomodulation treatment in photothrombosis‐induced stroke rats”

**DOI:** 10.1111/cns.14869

**Published:** 2024-07-16

Feng Y, Huang Z, Ma X, Zong X, Wu CY, Lee RH, Lin HW, Hamblin MR, Zhang Q. Activation of testosterone‐androgen receptor mediates cerebrovascular protection by photobiomodulation treatment in photothrombosis‐induced stroke rats. *CNS Neurosci Ther*. 2024 Feb;30(2): e14574.

Description of error: Erratum to “Figure 1I”. We noted that it showed the wrong testosterone/DAPI representative image in OGD group.

**FIGURE 1:**
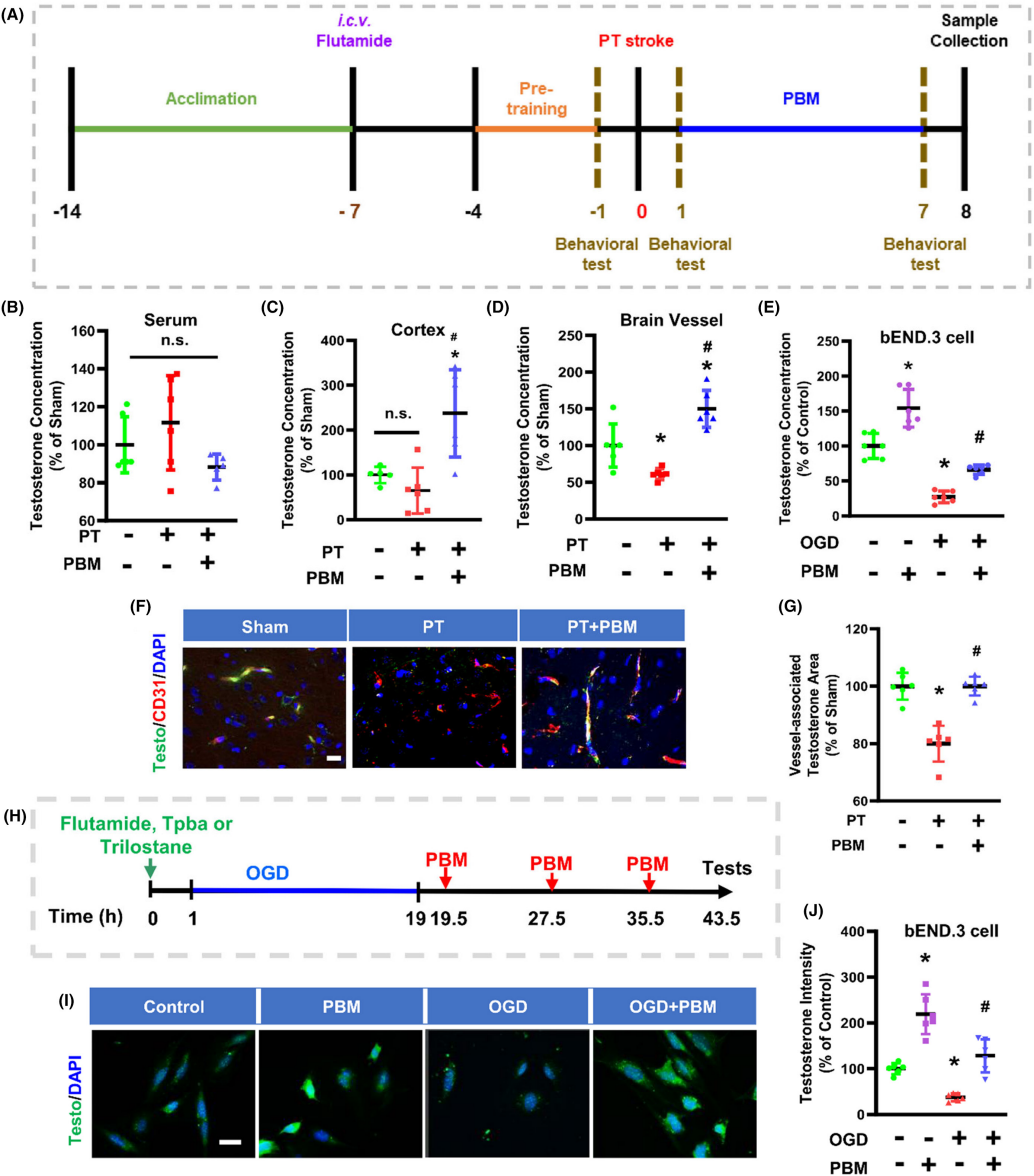
Photobiomodulation treatment (PBMT) increases vascular testosterone concentrations in photothrombosis (PT)‐stroke rats and OGD‐treated bEND.3 cells.

Please use the following corresponding figures.

We apologize for this error.

