# Supplementary material for: Activation of testosterone‐androgen receptor mediates cerebrovascular protection by photobiomodulation treatment in photothrombosis‐induced stroke rats
Source: CNS Neurosci Ther. 2024 Feb 8;30(2):e14574. doi: 10.1111/cns.14574 (PMC10851319; doi:10.1111/cns.14574)
Supplement: Supplementary file 1 — Figure S1. [file CNS-30-e14574-s001.docx]

**
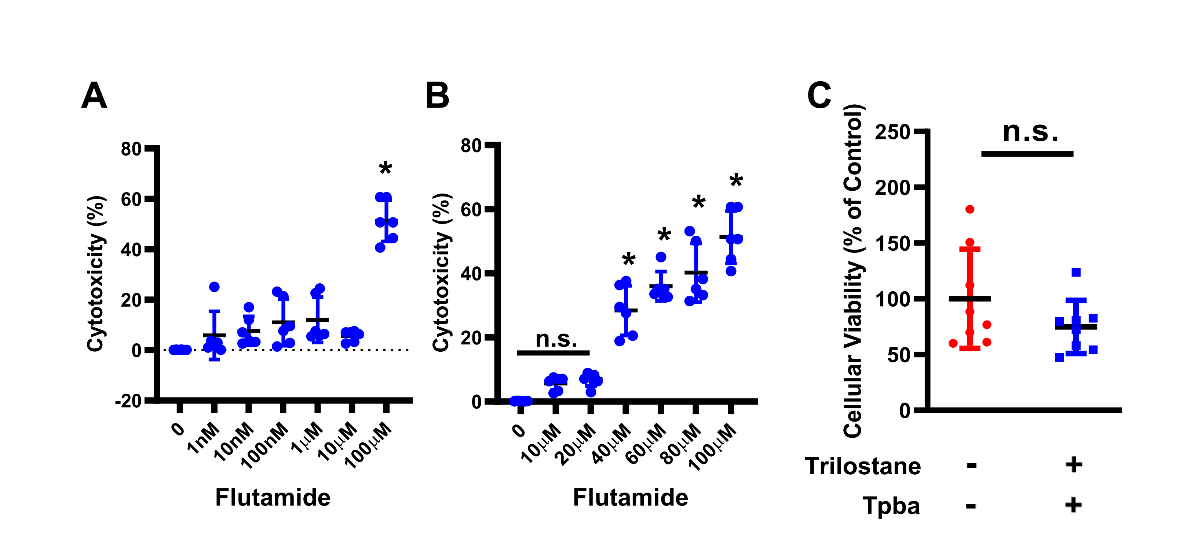
**

**Fig. S1 MTT assay was used to measure cellular cytotoxicity and viability in bend.3 cells. (A-B)** MTT assay was used to measure cytotoxicity in bEND.3 cells after treatment with flutamide at 1 nM to 100 μM concentrations. The data are expressed as mean ± SD (n = 5-6). One-way ANOVA followed by post-hoc Bonferroni’s test was used for analysis. **(C)** Treatment with both 25 μM Trilostane (3β-HSD1 inhibitor) and 10 μM Tpba (17β-HSD5 inhibitor) did not affect the cellular viability of bEnd.3 cells. The data are expressed as mean ± SD (n = 8). Data were analyzed by the student’s t-test. “n.s.” indicates no significant difference. * indicates P < 0.05 vs Control group.


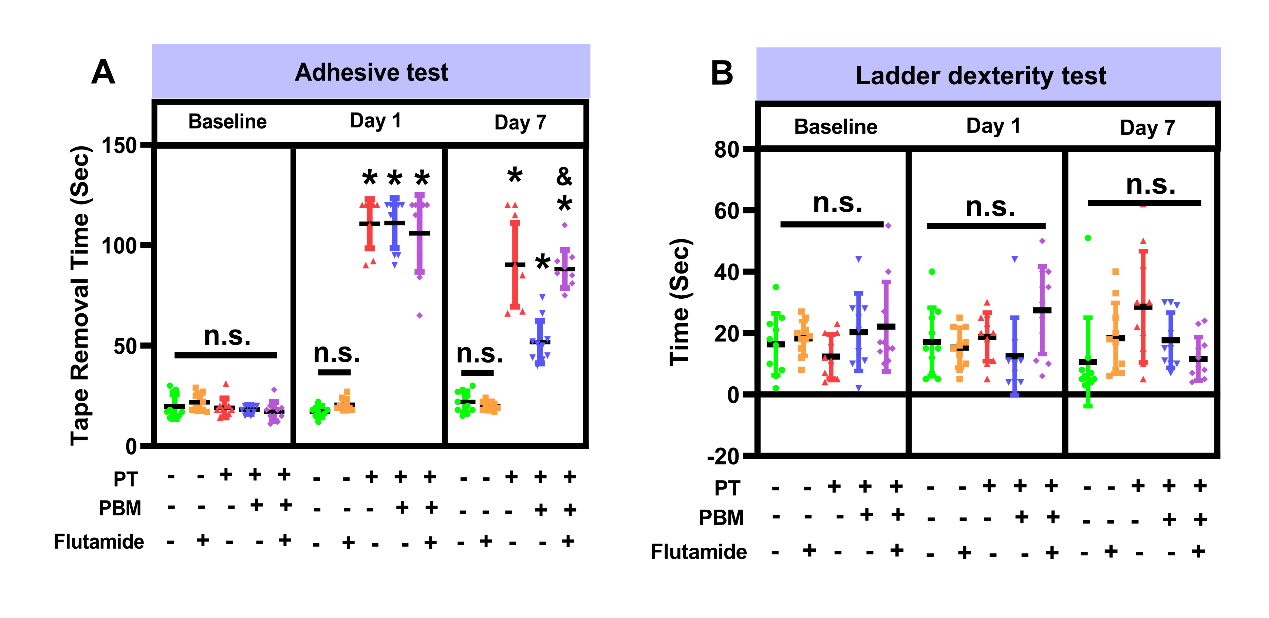


**Fig. S2 PBMT alleviated behavioral deficits in PT-stroke rats. (A)** The adhesive removal test was performed to test forepaw somatosensory activity. **(B)** The total time of the ladder dexterity test were performed to investigate motor coordination. The data are expressed as mean ± SD (n = 10). One-way ANOVA followed by post-hoc Bonferroni’s test was used for analysis. * indicates P < 0.05 vs Sham group; # indicates P < 0.05 vs PT-stroke group; & indicates P < 0.05 vs PT+PBM group. “n.s.” indicates no significant difference (P > 0.05).
